# Predictive Power of Pharyngolaryngeal Secretion Accumulations for Penetration and Aspiration in Head and Neck Cancer Patients

**DOI:** 10.1007/s00455-024-10801-3

**Published:** 2025-02-13

**Authors:** J. Hötzel, E. Zaretsky, A. Goeze, C. Hey

**Affiliations:** Department of Phoniatrics and Pediatric Audiology, Marburg University Hospital, Philipps University of Marburg, Baldingerstr. 1, 35032 Marburg, Germany

**Keywords:** Dysphagia, Head and neck cancer, Penetration-aspiration scale, Secretion severity rating scale, Swallowing disorders

## Abstract

Pharyngolaryngeal secretions, penetration, and aspiration belong to the cardinal symptoms of swallowing disorders in head and neck cancer patients. In order to provide safe swallowing diagnostics, it is of interest whether pharyngolaryngeal secretion accumulations can be used as a predictor for penetration and aspiration of food and liquids. This study analyzed the predictive power of pharyngolaryngeal secretion accumulations for the penetration and aspiration in head and neck cancer patients. Between 2016 – 2024, 403 head and neck cancer patients underwent swallowing diagnostics via Fiberoptic Endoscopic Evaluation of Swallowing (FEES). Pharyngolaryngeal secretions were graded with the secretion severity rating scale (SSRS) by Murray et al., penetration and aspiration with the penetration-aspiration scale (PAS) by Rosenbek et al. Both scales were cross-tabulated and correlated with each other. Possible influencing factors on PAS were analyzed by Spearman’s correlations and Kruskal–Wallis-*H*-tests: patients’ age, tumor stage and site, type of the oncological therapy, and time of examination. A linear regression and a Categorical Principal Components Analysis were utilized for the same purpose. Higher SSRS and PAS values tended to co-occur. In univariate calculations, the tumor stage and time of examination showed significant correlations with PAS. In multivariate analyses, the tumor stage, patients’ age, but above all the SSRS were shown to be significant influencing factors on PAS. Pharyngolaryngeal secretion accumulations can be used for the prediction of aspiration of food and liquids and thus for the improvement of safety of the swallowing diagnostics via FEES.

**Supplementary Information:**

The online version contains supplementary material available at 10.1007/s00455-024-10801-3.

## Introduction

Swallowing disorders are common in patients with head and neck cancer and occur in 8 – 70% of those affected [1, 2]. They result from the invasive tumor itself, but also from the tumor resection and/or (chemo)radiation. Swallowing disorders can lead not only to the reduced quality of life [3–5] but also to the aspiration [6, 7], malnutrition [8], and dehydration [9]. Therefore, they can influence significantly the oncological outcome [10] in many ways including the increased morbidity and mortality [11]. Also, aspiration and malnutrition, as consequences of the oropharyngeal dysphagia, increase the costs of the oncological therapy [12, 13].

A high prevalence rate of the oropharyngeal dysphagia, reduced quality of life, and the socio-economic burden on the health system and society underline the need of systematic assessment of the swallowing ability in patients with head and neck cancer. The assessment should aim not only to detect a swallowing disorder at an early stage but also to determine its severity. This is frequently done by the categorization of severity of the penetration and aspiration.

The assessment of accumulations of pharyngolaryngeal secretions, another cardinal symptom of the oropharyngeal dysphagia, can also be used for this purpose. These secretions provide information on the swallowing strength and frequency [14]. One of the key benefits of this approach is that it allows to draw important conclusions regarding swallowing functions without putting patients at risk. This is of particular importance for patients running a high risk of aspiration, such as those with head and neck cancer.

Pharyngolaryngeal secretion accumulations can be detected by the instrumental swallowing diagnostics via FEES (Fiberoptic Endoscopic Evaluation of Swallowing [15]). The assessment and graduation of pharyngolaryngeal secretion accumulations are carried out during the anatomical-physiological examination that is normally performed prior to the direct examination of swallowing. A uniform documentation of secretion accumulations can be performed by standardized and validated scales such as the four-point secretion severity rating scale (SSRS) by Murray et al. [16]. This scale focuses on the location of secretion accumulations in the hypopharynx and larynx.

Some studies showed considerable associations between pharyngolaryngeal secretion accumulations and dysphagia, aspiration as well as such dysphagia-related diagnoses as aspiration pneumonia in patients with various etiologies. Printza et al. [17] detected a strong correlation between pharyngeal secretions and aspirator status in a small group of patients with amyotrophic lateral sclerosis. Ota et al. [18] showed that higher SSRS values were associated with the swallowing dysfunction and a higher incidence of pneumonia in stroke patients. Miles et al. [19] reported a moderate correlation between the New Zealand Secretion Scale (NZSS) [20] and the penetration-aspiration scale (PAS) [21] as well as a considerable predictive power of NZSS for pneumonia in a mixed sample of patients with various etiologies (stroke, brain injuries, head and neck cancer, spinal injury etc.). Ponfick et al. [22] demonstrated that the improvement of SSRS grades in a small group of polyneuropathy patients was associated with the improvement of PAS grades for saliva, puree, and liquids. The Dynamic Imaging Grade of Swallowing Toxicity (DIGEST), an ordinal scale used to grade the severity of pharyngeal dysphagia [23], was shown to be moderately associated with the five-point Secretion severity scale [24] in head and neck cancer patients [25]. Kuo et al. [26] found a strong association between SSRS and PAS in a mixed sample of dysphagic patients. However, to our knowledge, no studies exist that scrutinized the predictive power of SSRS for PAS in a large sample of head and neck cancer patients.

Possible associations between pharyngolaryngeal secretion accumulations and penetration or aspiration can interact with a number of other influencing factors including patients’ and tumor characteristics. Thus, PAS grades of head and neck cancer patients are associated with the presence of sarcopenia [27], time of examination (assessment timing), type of the oncological therapy [28], patients’ age, tumor stage, pharyngeal constriction ratio, bolus clearance ratio [29], tumor location [30], lower preoperative serum albumin, the presence of diabetes [31] etc. Because multivariate analyses cannot cope with a large number of possible influencing factors, at least most well-known ones such as patients’ age and tumor stage should be considered in the calculations on the predictive power of SSRS for PAS.

Therefore, the aim of the present study was to analyze the predictive power of pharyngolaryngeal secretion accumulations, along with some other patients’ characteristics, for penetration and aspiration in a large sample of head and neck cancer patients. If the predictive power of secretion accumulations is high enough, their assessment can be used for the improvement of safety of the FEES diagnostics, especially in patients with an unknown aspiration risk.

It was hypothesized that pharyngolaryngeal secretion accumulations, quantified by SSRS, are predictive of a higher risk of penetration and/or aspiration, quantified by PAS, irrespective of patients’ age, tumor stage, tumor site, type of the oncological therapy, or time of examination.

## Methods

A total of 403 head and neck cancer patients, 303 (75.2%) male and 100 (24.8%) female, mean age 63.2 ± 9.8 years (span 18 – 87 years), were recruited for this prospective, cross-sectional study between March 2016 and April 2024 in the Department for Phoniatrics and Pediatric Audiology in the Marburg University Hospital (state of Hesse, Germany). In Germany, some of such departments focus on dysphagia assessment and treatment in head and neck cancer patients.

Inclusion criteria were defined as (a) patients with head and neck cancer (oral cavity or oropharynx or hypopharynx or larynx tumors), (b) tumor stages I – IV according to the UICC staging system (UICC = Union international contre le cancer), (c) pre-treatment or tumor resection and/or chemo(radio)therapy, (d) age 18 – 99 years, (e) no laryngectomy, and (f) no neurological diseases that can result in dysphagia (e.g., Korsakoff syndrome, stroke).

All patients undergoing head and neck cancer treatment in the Marburg University Hospital were routinely contacted by speech-language pathologists of our department for the dysphagia assessment. Among these patients, those fulfilling inclusion criteria were invited to participate in the study. Patients’ decision in favor of or against the study participation did not influence the dysphagia assessment modalities. All patients’ characteristics that were utilized for statistical analyses in the present study were routinely documented in medical reports. Therefore, study participants did not have to undergo any additional medical examinations or to fill out any additional questionnaires or forms.

Patients’ characteristics, including patients’ age, stage and site of tumor, type of the oncological therapy as well as time of examination, are listed in Table 1.

**Table 1:** Patients’ characteristics (*N* = 403)

	*n*	*%*
Age (years)		
18 – 49	26	6.5
50 – 59	119	29.5
60 – 69	166	41.2
70 – 87	92	22.8
Tumor stage (UICC†)		
I	40	9.9
II	75	18.6
III	66	16.4
IV	222	55.1
Tumor site		
Oral cavity	130	32.3
Oropharynx	169	41.9
Hypopharynx / larynx	104	25.8
Oncological therapy		
Pre-treatment	32	7.9
Primary surgery	161	40.0
Primary surgery and adjuvant chemo(radio)therapy	100	24.8
Primary chemo(radio)therapy	110	27.2
Time of examination		
Pre-treatment	32	7.9
Short after tumor resection^††^	135	33.5
1 month after treatment onset	161	40.0
3 months after treatment onset	47	11.7
6 months after treatment onset	16	4.0
12 months after treatment onset	12	3.0

^†^Union International Contre le Cancer; ††usually one or two weeks after surgery

The study was conducted in accordance with the Declaration of Helsinki (1975) in its revised version as well as with the written consent of the ethics committee of the Marburg University Hospital (# AZ 63/15).

FEES (according to the Langmore standard [15]) was performed and recorded digitally by a phoniatrician with more than 15 years of experience in FEES. According to this standard, the FEES procedure is divided into three parts: (1) anatomic-physiologic assessment, (2) direct examination of swallowing of food and liquids, and (3) therapeutic maneuvers and their effects.

Baseline pharyngolaryngeal secretions without bolus were analyzed during the anatomic-physiologic assessment. The penetration and aspiration were quantified during the direct examination of swallowing of food and liquids by means of three consistencies. For safety reasons, FEES was adapted to the patients’ swallowing ability. It started with 2 ml of water followed by increasing volumes (5, 10, and 20 ml) and, if possible, by puree (2 and 5 ml) as well as a bite of cookie. The examination was discontinued if aspiration occurred. The FEES procedure took around 10 to 15 min.

For this examination, a transnasal flexible endoscope 11,101 RP2 (Karl Storz GmbH, Germany) was used. The digital recording was conducted by an ENT video endoscopy system EndoStrob-DX (Xion medical GmbH, Germany) with 29.8 frames per second.

FEES recordings were analyzed by two dysphagia experts with more than 20 and 15 years of experience in FEES. The grading of the accumulation of pharyngolaryngeal secretions within the hypopharynx and larynx was performed according to the four-point secretion severity rating scale by Murray et al. [16] (see Table S1 in Online Resource 1). The eight-point penetration-aspiration scale by Rosenbek et al. [21] (see Table S2 in Online Resource 1) was used to grade the depth of penetration and aspiration, patients’ reactions to penetration and aspiration as well as effectivity of clearing attempts.

The inter-rater reliability of the SSRS and PAS grading was analyzed for a subsample of 40 FEES records that were selected randomly from 403 videos (10% of the sample). These 40 videos were analyzed by both raters who were blinded for each other’s judgments. Judgments of both raters were compared by a weighted kappa for ordinal data. The inter-rater reliability can be considered excellent for PAS (*κ* = 0.92, 85.0% agreement) and good for SSRS (*κ* = 0.70, 77.5% agreement).

### Statistical Analysis

First, the absolute and relative frequency of pharyngolaryngeal secretions, penetration, and aspiration was determined. Next, SSRS and PAS were cross-tabulated, including Chi-Square calculation, to assess their association patterns. For this analysis, PAS was trichotomized into (1) no penetration and no aspiration (PAS value 1), (2) penetration (PAS values 2 – 5), and (3) aspiration (PAS values 6 – 8) according to the categorization specified by Rosenbek et al. [21], while the Murray scale (SSRS) was used unchanged.

The strength of association between SSRS and PAS was quantified by a Spearman’s correlation.

Associations between potential influencing factors and both scales (SSRS and PAS) were calculated by univariate methods including Spearman’s correlation for the tumor stage, patients’ age, and time of examination as well as Kruskal–Wallis-*H*-test for the tumor site and type of the oncological therapy.

Next, PAS values entered a linear regression as dependent variable. Patients’ age in years, their tumor stage and site, type of the oncological therapy, time of examination as well as SSRS were utilized as independent variables to analyze the predictive power of SSRS for PAS results under consideration of other potential influencing factors. The percentage of the variance explained by the chosen independent variables was quantified by the corrected *R*^2^.

The same variables entered a Categorical Principal Components Analysis (CATPCA) for the visualization of their associations. CATPCA is a multivariate statistical method that can process categorical, ordinal, and metrical variables. In CAPTCA visualizations, metrical or ordinal factors are depicted as lines, categorical ones as dots (each dot stands for one category). If two ordinal or metrical variables (depicted as lines) build a right angle, there is no correlation between them. Sharp angles and overlapping lines mean high correlations. For categorical variables, a large distance from the respective dot to other dots or lines means a weak (or no) association, a short distance means a strong association. The internal consistency coefficient (Cronbach's Alpha) was calculated to assess the quality of the model.

Finally, the predictive power of SSRS for PAS was quantified in the calculations of sensitivity, specificity, positive and negative predictive values. Both scales were dichotomized (“pass/fail”) and cross-tabulated for this purpose. All possible dichotomizations of SSRS were tried out as cut-off criteria for the prediction of (a) aspiration (PAS ≥ 6) and (b) penetration (PAS 2 – 5).

## Results

Out of 403 head and neck cancer patients, there were no pharyngolaryngeal secretions in 223 patients (55.3%) and neither penetration nor aspiration in 103 (25.6%) patients. Pharyngolaryngeal secretions were detected in 180/403 (44.7%) patients: with accumulations in the valleculae or pyriform sinus (SSRS value = 1) in 85/403 (21.1%) patients, transient in the laryngeal inlet (SSRS value = 2) in 71/403 (17.6%) patients, and constant (SSRS value = 3) in 24/403 (6.0%) patients. Penetration was found in 177/403 (43.9%) patients, aspiration in 123/403 (30.5%) patients, with silent aspiration in 44/403 (10.9%) patients.

The frequency distribution of both scales (SRSS and PAS) is described in the following cross-table (see Table 2). Lower SSRS values tended to co-occur with lower PAS values, higher SSRS values with higher PAS values (*χ*^2^_(6)_ = 109.21, *p* < 0.001).

**Table 2:** Absolute and relative frequency of SSRS and PAS values: cross-table

			Categorized penetration-aspiration scale (PAS)†			
			1	2 – 5	6 – 8	Total
Secretion severity rating scale (SSRS)		0	88 (39.5%)	108 (48.4%)	27 (12.1%)	223 (100.0%)
		1	13 (15.3%)	37 (43.5%)	35 (41.2%)	85 (100.0%)
		2	2 (2.8%)	26 (36.6%)	43 (60.6%)	71 (100.0%)
		3	0 (0.0%)	6 (25.0%)	18 (75.0%)	24 (100.0%)
		Total	103 (25.6%)	177 (43.9%)	123 (30.5%)	403 (100.0%)

^†^PAS categorized as “1=no penetration or aspiration; 2 – 5=penetration; 6 – 8=aspiration”

The Spearman’s correlation between SSRS and PAS amounted to *ρ* = 0.525, *p* < 0.001, and can be considered high according to Cohen [32]. Again, higher SSRS and PAS values co-occurred.

Univariate statistics on associations of both scales (SSRS and PAS) with patients’ characteristics are reported in Table 3 (Spearman’s correlations and Kruskal–Wallis-*H*-tests), respective median SSRS and PAS values in Table 4. Higher UICC tumor stages were associated with higher values on both scales. Time of examination closer to the oncological therapy was associated with higher PAS values. Other variables – patients’ age, tumor site, and type of the oncological therapy – did not yield significant results in univariate analyses.

**Table 3:** Associations between patients’ characteristics and (a) secretion severity rating scale, (b) penetration-aspiration scale: Spearman’s correlations (*ρ*), Kruskal–Wallis-*H*-tests (*χ*^2^)

	Secretion severity rating scale	Penetration-aspiration scale
Patients’ age	*ρ* = -.026	*ρ* = .092
Tumor stage (UICC†)	*ρ* = .185***	*ρ* = .275***
Tumor site	*χ*^2^_(2)_ = 4.42	*χ*^2^_(2)_ = 3.60
Oncological therapy	*χ*^2^_(3)_ = 0.34	*χ*^2^_(3)_ = 4.31
Time of examination	*ρ* = -.066	*ρ* = -.122*

***p < .001, *p < .05^; †^Union International Contre le Cancer

**Table 4:** Medians and interquartile ranges (IR) of the secretion severity rating scale (SSRS) and penetration-aspiration scale (PAS) depending on patients’ characteristics

	Median SSRS ± IR	Median PAS ± IR
Patients’ age (years)		
18 – 49	0.5 ± 1.0	2.5 ± 5.0
50 – 59	0.0 ± 1.0	3.0 ± 5.0
60 – 69	0.0 ± 2.0	3.0 ± 4.0
70 – 87	0.0 ± 1.0	3.0 ± 6.0
Tumor stage (UICC†)		
I	0.0 ± 0.0	1.5 ± 2.0
II	0.0 ± 1.0	2.0 ± 4.0
III	0.0 ± 2.0	3.0 ± 5.0
IV	1.0 ± 2.0	4.0 ± 5.0
Tumor site		
Oral cavity	0.0 ± 1.0	3.0 ± 5.0
Oropharynx	0.0 ± 2.0	3.0 ± 5.0
Hypopharynx / larynx	0.0 ± 2.0	3.0 ± 5.0
Oncological therapy		
Pre-treatment	0.0 ± 1.0	3.0 ± 4.0
Primary surgery	0.0 ± 2.0	3.0 ± 6.0
Primary surgery and adjuvant chemo(radio)therapy	0.0 ± 1.0	3.0 ± 5.0
Primary chemo(radio)therapy	0.0 ± 1.0	2.5 ± 3.0
Time of examination		
Pre-treatment	0.0 ± 1.0	2.5 ± 4.0
Short after tumor resection^††^	1.0 ± 2.0	5.0 ± 5.0
1 month after treatment onset	0.0 ± 2.0	3.0 ± 3.0
3 months after treatment onset	0.0 ± 1.0	2.0 ± 4.0
6 months after treatment onset	0.0 ± 1.0	3.5 ± 4.0
12 months after treatment onset	0.0 ± 1.0	3.0 ± 3.0

^†^Union International Contre le Cancer; ††usually one or two weeks after surgery

The linear regression (see Table 5) accounted for 31.2% of variance of PAS values (corr. *R*^2^ = 0.312). According to ANOVA, the chosen regression model was highly significant: *F*_(6, 402)_ = 31.4, *p* < 0.001.

**Table 5:** Possible influencing factors on the penetration-aspiration scale: A linear regression with patients’ characteristics as independent variables (method “enter”)

	Regression coefficients (*β*)	*T*
Secretion severity rating scale	1.30	11.68***
Patients’ age	0.03	2.64**
Tumor stage (UICC^†^)	0.42	4.07***
Tumor site	− 0.08	− 0.58
Oncological therapy	0.03	0.24
Time of examination	− 0.16	− 1.58
Constant	0.29	0.36

***p < .001, **p < 0.01, *p < .05; ^†^Union International Contre le Cancer

According to non-standardized regression coefficients in Table 5, lower patients’ age, UICC tumor stages I and II, and SSRS values 0 and 1 were significantly associated with better PAS results. Type of the oncological therapy, tumor site, and time of examination did not influence the occurrence of penetration or aspiration significantly.

CATPCA is visualized in Figure S1 (see Online Resource 1). Cronbach's Alpha amounted to 0.775 (good quality of the model). PAS values 6 – 8 showed a strong association with SSRS values 2 – 3. Also, high PAS values tended to co-occur with (a) UICC tumor stages III and IV, (b) tumor resection with adjuvant chemo(radio)therapy, (c) time of examination before therapy or short after tumor resection as well as with (d) oropharynx carcinomas. However, associations with all these variables except SSRS were weak. SSRS grade 0 is located close to PAS grades 1 and 2, that is, a “pass” result in SSRS (no pharyngolaryngeal secretion accumulations) is associated with no or minimal penetration.

Results of the cross-tabulation of dichotomized SSRS and PAS can be found in Table 6. Three different cut-off values of SSRS were tried out for the prediction of the aspiration (PAS 6 – 8) and penetration (PAS 2 – 5). In the latter case, the sample size decreased from *N* = 403 to *n* = 280 because patients with PAS grades 6 – 8 were excluded.

**Table 6:** Secretion severity rating scale (SSRS) with different cut-off values for the prediction of the penetration-aspiration scale (PAS): sensitivity, specificity, positive (PPV) and negative predictive values (NPV)

Cut-off values	SSRS	Patients in non-aspiration group (PAS ≤ 5)	Patients in aspiration group (PAS ≥ 6)	Sensitivity (%)	Specificity (%)	PPV (%)	NPV (%)
1	0	196/223 (87.9%)	27/223 (12.1%)	78.1	70.0	53.3	87.9
	1 – 3	84/180 (46.7%)	96/180 (53.3%)				
2	0 – 1	246/308 (79.9%)	62/308 (20.1%)	49.6	87.9	64.2	79.9
	2 – 3	34/95 (35.8%)	61/95 (64.2%)				
3	0 – 2	274/379 (72.3%)	105/379 (27.7%)	14.6	97.9	75.0	72.3
	3	6/24 (25.0%)	18/24 (75.0%)				

Cut-off values	SSRS	Patients in non-penetration group (PAS 1)	Patients in penetration group (PAS 2 – 5)	Sensitivity (%)	Specificity (%)	PPV (%)	NPV (%)
1	0	88/196 (44.9%)	108/196 (55.1%)	39.0	85.4	82.1	44.9
	1 – 3	15/84 (17.9%)	69/84 (82.1%)				
2	0 – 1	101/246 (41.1%)	145/246 (48.9%)	18.1	98.1	94.1	41.1
	2 – 3	2/34 (5.9%)	32/34 (94.1%)				
3	0 – 2	103/274 (37.6%)	171/274 (62.4%)	3.4	100.0	100.0	37.9
	3	0/6 (0.0%)	6/6 (100.0%)				

## Discussion

Pharyngolaryngeal secretion accumulations, penetration, and aspiration are considered cardinal symptoms of the oropharyngeal dysphagia and therefore require special attention in head and neck cancer patients. In the present study, about one in two patients had pharyngolaryngeal secretion accumulations and almost one in four showed at least transient secretion accumulations in the laryngeal inlet. Three quarters of head and neck cancer patients also demonstrated penetration or aspiration in the swallowing function test with food and liquids. Pharyngolaryngeal secretion accumulations, penetration, and aspiration showed a clear association: the higher the SSRS value, the higher the PAS value, and also the percentage of patients with penetration or aspiration. According to multivariate analyses, the severity of penetration and aspiration was influenced by the patients’ age, tumor stage and, above all, by the occurrence of pharyngolaryngeal secretion accumulations. These findings highlight the importance of the documentation and graduation of pharyngolaryngeal secretion accumulations as a possible predictor for penetration and aspiration in head and neck cancer patients.

Although there are some studies that reported the prevalence of pharyngolaryngeal secretion accumulations, the findings were very heterogeneous. This can be attributed to differences in the study design, diagnostic procedures, sample sizes or patient populations. Also, most studies on pharyngolaryngeal secretion accumulations focused on neurological patients or those who underwent long-term ventilation [33, 34]. Furthermore, predominantly other scales were used, such as those by Donzelli et al. [24] or Miles and Hunting [20]. These scales consider further factors (e.g., patients’ reaction) to grade the accumulation of secretions.

Even in case of SSRS the prevalence of pharyngolaryngeal secretion accumulations varies significantly, as can be seen in the following three studies. Kuo et al. [26] reported pharyngolaryngeal secretion accumulations in 60% of head and neck cancer patients (cf. 45% in the present study). Shapira-Galitz et al. [33] found secretion accumulations in 39% of their mixed sample including head and neck cancer patients as the largest subgroup. Yamaguchi et al. [34] identified secretion accumulations in 77% of their small sample of head and neck cancer patients.

There is also a wide range of prevalence values on penetration and aspiration depending on many factors such as tumor site or size as well as type of the oncological therapy [35]. The list of possible influencing factors on the prevalence can be continued with the sample size, examination instruments, and assessment timing. The latter refers especially to head and neck cancer patients who undergo a radiotherapy and may develop penetration and aspiration due to late toxicity [36–38]. Discrepancies in study results can also be partially attributed to a considerable intra-individual variability of PAS values shown in the study by Hedström et al. [39], that is, the same patient can show different PAS results with the same bolus size at the same point in time.

After the onset of the oncological therapy, the prevalence of penetration ranges between 7 – 75% of head and neck cancer patients [40], that of aspiration between 5 – 78% [29] (cf. 44% and 31% respectively in the present study). PAS values before therapy onset are much lower, e.g., aspiration in 13% of patients in the study by Gupta et al. [35], but in the present study most patients were tested after the therapy onset.

In the univariate analyses of possible influencing factors on SSRS and PAS, no clear associations between three patients’ characteristics (patients’ age, tumor site, type of the oncological therapy) and these two scales were found in the present study. The only significant result in univariate calculations for both scales was demonstrated by the UICC tumor stage, although Spearman’s correlations can be considered low. Higher values on both scales were associated with higher tumor stages (III and IV). This finding was confirmed by the linear regression. However, the highest predictive power for PAS results in this multivariate analysis was demonstrated not by UICC tumor stages but by SSRS, that is, patients with more pharyngolaryngeal secretion accumulations tended to show penetration and aspiration more frequently. Also, the patients’ age that yielded only a marginally significant result in the univariate analysis (*p* = 0.065) did demonstrate a certain predictive power (*p* = 0.010) for penetration and aspiration under consideration of other possible influencing factors in the linear regression. Older patients tended to score higher on PAS.

Examinations that were closer to the onset of the oncological therapy resulted in higher PAS values in univariate calculations, which might indicate positive therapy results after the oncological treatment. However, the correlation was very low and this factor did not show a significant result in the regression.

According to the second multivariate analysis, CATPCA, patients with aspiration (PAS ≥ 6) tended to have pharyngolaryngeal secretion accumulations (SSRS 2 – 3), oropharynx carcinomas, UICC tumor stages III or IV, and they underwent a tumor resection with adjuvant chemo(radio)therapy. Dots representing PAS grades 6 – 8 are located very close to each other (in Figure S1) because they share common influencing factors. The same factors are associated with SSRS grades 1 – 3. However, CATPCA identifies even very weak tendencies that would not be statistically significant in other univariate or multivariate analyses.

Results of previous studies on possible influencing factors on PAS in head and neck cancer patients are inconsistent. Lehner et al. [7] reported for head and neck cancer patients prior to the onset of the oncological therapy that patients’ age did not influence PAS values, whereas tumor stage and site did (in a linear regression). Goeze et al. [6] found in a sample of head and neck cancer patients short after tumor resection a significant influence of their age and tumor stage on PAS, but no influence of tumor site (in a linear regression). Liou et al. [30] analyzed a sample of head and neck cancer patients after the oncological therapy (any type) and detected a significant influence of tumor site and patients’ age on PAS (in a logistic regression). Liu et al. [29] found a significant influence of patients’ age and tumor stage, but not of tumor site, on PAS in a sample of head and neck cancer patients after radiotherapy (in a logistic regression). However, in spite of such discrepancies, the direction of associations is usually the same, that is, patients of higher age and with higher UICC tumor stages tend to show worse PAS results. Tumor site represents the only exception because all tumor sites were described at least once as most strongly associated with higher PAS grades. Thus, according to Lehner et al. [7], hypopharynx/larynx tumors were associated with worst PAS values, according to Liou et al. [30] these were oropharynx tumors.

The correlation between SSRS and PAS in the present study was high but lower than in the study by Kuo et al. [26] (0.53 vs. 0.81). The latter study also focused on the predictive power of pharyngolaryngeal secretion accumulations for penetration and aspiration in dysphagic patients. However, Kuo et al. [26] did not provide any information on the patients’ characteristics such as tumor stage and site. Therefore, it is difficult to trace back these discrepancies in correlation coefficients to differences in the study design or sample characteristics. However, the sample of Kuo et al. [26] had another ratio of head and neck cancer patients with penetration and aspiration than the sample in the present study. Thus, Kuo et al. reported 92% of patients with penetration, whereas in the present study these were only 44%. Also, Kuo et al. had only 8% of patients with aspiration, whereas in the present study these were 31%, despite the same PAS categorizations.

In terms of test quality criteria, SSRS showed unsatisfactory sensitivity and positive predictive value for aspiration (PAS ≥ 6). Kuo et al. [26] also demonstrated insufficient quality criteria in the prediction of aspiration by means of dichotomized SSRS values. The maximization of sensitivity to 100% resulted in the study by Kuo et al. [26] in the too low specificity of 44%, whereas the maximization of specificity to 99% resulted in the too low sensitivity of 21%. Shapira-Galitz et al. [33] also utilized SSRS for the prediction of aspiration in a mixed sample of dysphagic patients. The quality criteria were, again, too low, with the sensitivity of 75% and the specificity of 65%. However, it should be considered that SSRS was not designated as a screening instrument for PAS and, therefore, very high test quality criteria were not expected.

Furthermore, SSRS did not deliver satisfactory cut-off criteria for the detection of PAS grades 2 – 5 (penetration) in the present study. Although the ratio of patients truly diagnosed as positive to all those who had positive test results (positive predictive value) was very high, the proportion of patients with a negative test result who truly do not have penetration or aspiration (negative predictive value) can be considered too low. The sensitivity was also insufficient.

The study population was predominantly male, with patients’ age over 60 years, tumor site in the oropharynx, and UICC stage IV. The sample can be considered representative because head and neck cancer patients’ characteristics are comparable to those in the German data of the Robert Koch Institute [41, 42]. These data demonstrate that German head and neck cancer patients are more often male than female, are usually above 60 years old and oropharynx is the most frequent tumor site.

One of limitations of the study was the statistical analysis of patients with hypopharynx and larynx tumors as one group due to low sample sizes. However, this approach can be found in other studies as well [6, 7], although these two tumor sites do not necessarily result in the same SSRS and PAS values. With 32 patients, the pre-treatment subgroup was much smaller than other categories in the variable “type of the oncological therapy”. This can also be considered a limitation of the study and might have influenced the result of the Kruskal–Wallis-*H*-test. Furthermore, it cannot be excluded that COVID-19 restrictions introduced some bias into the recruitment of patients (e.g., comparatively high UICC stages due to the prolonged diagnostics time during the pandemic). However, no studies exist that would show significant changes in the time of diagnostics of head and neck cancer patients in the Marburg University Hospital since 2020.

The study presented here is the first one that investigates the predictive power of pharyngolaryngeal secretion accumulations, quantified by SSRS, for penetration and aspiration of food and liquids, quantified by PAS, in a large sample of head and neck cancer patients. The large sample size might allow generalization of two main findings: first, a significant positive correlation between tumor stages and PAS values, second, a considerable predictive power of pharyngolaryngeal secretion accumulations for aspiration.

To conclude, laryngopharyngeal secretion accumulations can be used to a certain extent to predict the penetration and aspiration of food and liquids, thereby making FEES diagnostics safer in the interest of patient care.

## Supplementary Information

Below is the link to the electronic supplementary material.

Supplementary file1 (DOCX 869 KB)

## Data Availability

The data belong to the Marburg University Hospital and cannot be uploaded to any repositories due to ethical reasons.
