# Supplementary material for: Predictive Power of Pharyngolaryngeal Secretion Accumulations for Penetration and Aspiration in Head and Neck Cancer Patients
Source: Dysphagia. 2025 Feb 13;40(5):1055–63. doi: 10.1007/s00455-024-10801-3 (PMC12479604; doi:10.1007/s00455-024-10801-3)
Supplement: Supplementary file 1 — Supplementary file1 (DOCX 869 KB) [file 455_2024_10801_MOESM1_ESM.docx]

**Predictive power of pharyngolaryngeal secretion accumulations for penetration and aspiration in head and neck cancer patients**

[Author names: anonymized]

Journal: Dysphagia

Supplementary Data

Table S1: Secretion severity rating scale by Murray et al. [16]

| 0 | Most normal rating. No visible secretions anywhere in the hypopharynx or some transient bubbles visible in the valleculae and pyriform sinuses. These secretions were not bilateral or deeply pooled. |
| --- | --- |
| 1 | Any secretions evident upon entry or following a dry swallow in the channels surrounding the laryngeal vestibule that were bilaterally represented or deeply pooled. This rating would include cases where there is a transition in the accumulation of secretions during the observation segment. A subject could start with no visible secretions but accumulate secretions in an amount great enough to be bilaterally represented or deeply pooled. Likewise, a subject would be rated as a "1" if initially presenting with deeply pooled bilateral secretions and ending the observation segment with no visible secretions. |
| 2 | Any secretions that changed from a "1" rating to a "3" rating during the observation period. |
| 3 | Most severe rating. Any secretions seen in the area defined as the laryngeal vestibule. Pulmonary secretions were included if they were not cleared by swallowing or coughing at the close of the segment. |

Table S2: Penetration-aspiration scale by Rosenbek et al. [21]

| 1 | Material does not enter the airway. |
| --- | --- |
| 2 | Material enters the airway, remains above the vocal folds, and is ejected from the airway. |
| 3 | Material enters the airway, remains above the vocal folds, and is not ejected from the airway. |
| 4 | Material enters the airway, contacts the vocal folds, and is ejected from the airway. |
| 5 | Material enters the airway, contacts the vocal folds, and is not ejected from the airway. |
| 6 | Material enters the airway, passes below the vocal folds, and is ejected into the larynx or out of the airway. |
| 7 | Material enters the airway, passes below the vocal folds, and is not ejected from the trachea despite effort. |
| 8 | Material enters the airway, passes below the vocal folds, and no effort is made to eject. |

Figure S1. Categorical Principal Components Analysis (CATPCA) for variables 1) oncological therapy, 2) patients’ age, 3) penetration-aspiration scale, 4) secretion severity rating scale, 5) time of examination, 6) tumor site, 7) tumor stage (UICC^†^)


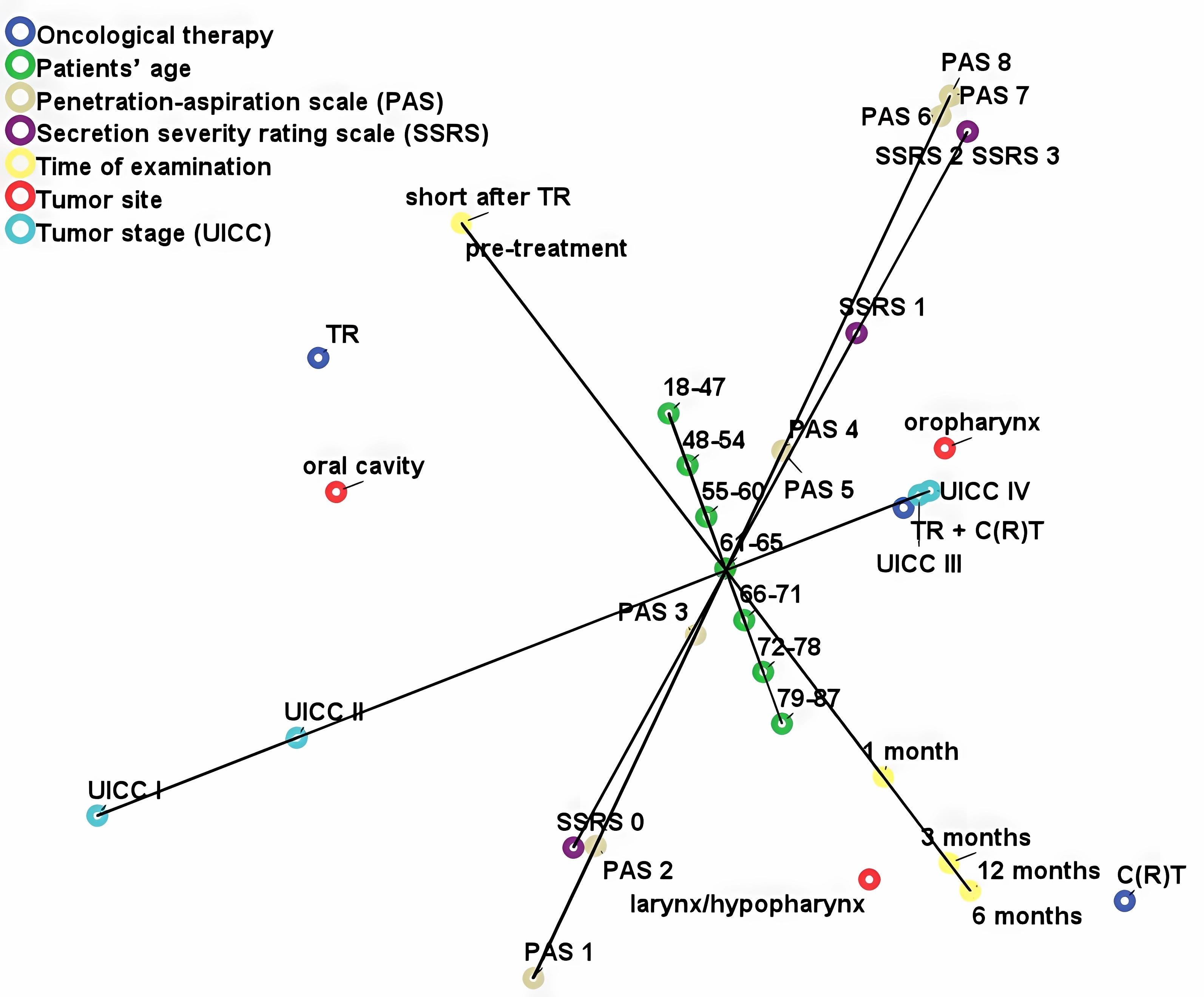


^† Union International Contre le Cancer, C(R)T chemo(radio)therapy, TR tumor resection^
